# Sarcopenia defined with L3-SMI is an independent predictor of survival in male patients with ARLD in mainland China

**DOI:** 10.3389/fnut.2023.1238433

**Published:** 2023-09-15

**Authors:** Yu Zhang, Liangui Wei, Chunyan Chang, Fangfang Duan, Min Quan, Song Yang

**Affiliations:** ^1^Department of Hepatology, Beijing Ditan Hospital of Capital Medical University, Beijing, China; ^2^Department of Radiology, Beijing Ditan Hospital of Capital Medical University, Beijing, China; ^3^Department of Hepatology, The Fourth People’s Hospital of Qinghai Province, Xining, China

**Keywords:** sarcopenia, alcohol-related liver disease, L3-SMI, prognosis, survival

## Abstract

**Background:**

The burden of alcohol-related liver disease (ARLD) is increasing in China. Patients with ARLD are more likely to have comorbid sarcopenia, which may impair their survival. This study aimed to evaluate the relationship between the prognoses of patients with ARLD and sarcopenia, identified using the skeletal muscle index at the third lumbar vertebra level (L3-SMI).

**Methods:**

Hospitalized patients with ARLD were retrospectively enrolled between 2015 and 2018 and followed up for 24 months to evaluate their survival profiles. Cox proportional hazards regression models were used to estimate patient survival factors. A receiver operating characteristic curve was created to identify the cut-off point of the L3-SMI for predicting the prognoses of Chinese patients with ARLD.

**Results:**

The study enrolled 168 male patients with ARLD who were followed-up for 24 months or until a study endpoint was met. The overall L3-SMI in patients with ARLD was 42.61 ± 9.15 cm^2^/m^2^, and 42.86% (72/168) of patients with ARLD were comorbid with sarcopenia. The overall survival in patients with ARLD was 77.38% at 24 months. The survival rate of patients with sarcopenia was lower than that of patients without sarcopenia (66.67% vs. 85.42%, *p* = 0.004). Multiple Cox regression analysis showed that sarcopenia, abstinence, and baseline creatinine level were independent prognostic factors of 24-month survival with hazard ratios (95% confidence intervals) of 2.022 (1.025–3.991), 0.275 (0.122–0.617), and 1.018 (1.008–1.027), respectively. The cut-off value of the L3-SMI for predicting 24-month survival was 40.0 cm^2^/m^2^ for male patients with ARLD.

**Conclusion:**

Sarcopenia is an independent mortality risk factor in male patients with ARLD in mainland China. Early diagnosis and intervention of sarcopenia are important for optimizing the management of patients with ARLD.

## 1. Introduction

Alcohol-related liver disease (ARLD) is the leading cause of liver disease, accounting for 50% of all liver disease-related deaths worldwide (1). The incidence of ARLD and related deaths is also on the rise in China (2). Multiple studies have shown that many factors are associated with the prognoses of patients with ARLD, including abstinence, baseline cirrhosis, and renal dysfunction (3–5). Recently, the relationship between sarcopenia and the prognoses of patients with liver disease has attracted increasing attention (6). Sarcopenia is a common syndrome in patients with chronic liver disease, and is characterized by the progressive and systemic loss of skeletal muscle mass and strength. Studies have shown that sarcopenia is a key risk factor for the survival of patients with cirrhosis (7). The incidence of sarcopenia in patients with ARLD is greater than that in patients with other causes of liver disease. The prevalence of sarcopenia in patients with ARLD is 37–50%, significantly affecting the survival of patients with ARLD (7). However, the relationship between sarcopenia and survival in male patients with ARLD in mainland China remains unclear.

Regarding the diagnosis criteria of sarcopenia, the European Working Group on Sarcopenia in the Elderly (EWGSOP) recommended using handgrip strength, knee flexion/extension strength, usual gait speed, and medical imageology to assess muscle mass and strength (8). According to these guidelines, a diagnosis of sarcopenia can be made in males with a handgrip strength of <30 kg and in females with <20 kg. However, there are some confounding factors; for instance, handgrip strength correlated with leg strength, making the measures vulnerable to error and questionable for routine use. Computed tomography (CT), which is regarded as the gold standard for analyzing body composition, is more accurate (9, 10). In particular, the skeletal muscle index at the third lumbar vertebra (L3-SMI), measured using CT (11, 12), has been validated for evaluating total skeletal muscle and is used for the diagnosis of sarcopenia (13–15). Due to differences in ethnicity, dietary habits, and physical activity, the criteria for L3-SMI in Asian populations is significantly different to that of other populations, such as those from the United States and Europe [45.4 cm^2^/m^2^ in men and 34.4 cm^2^/m^2^ in women from the United States (16), and 43.1 cm^2^/m^2^ in men and 32.7 cm^2^/m^2^ in women from the Netherlands (17)]. Recently Kong et al. (12) reported in China that the cut-off value for the L3-SMI for the diagnosis of sarcopenia was 40.2 cm^2^/m^2^ in male patients and 31.6 cm^2^/m^2^ in female patients. The current study aimed to analyze the relationship between the prognoses of male patients with ARLD and sarcopenia, as defined using the L3-SMI.

## 2. Methods

### 2.1. Patients and study design

**Figure 1:**
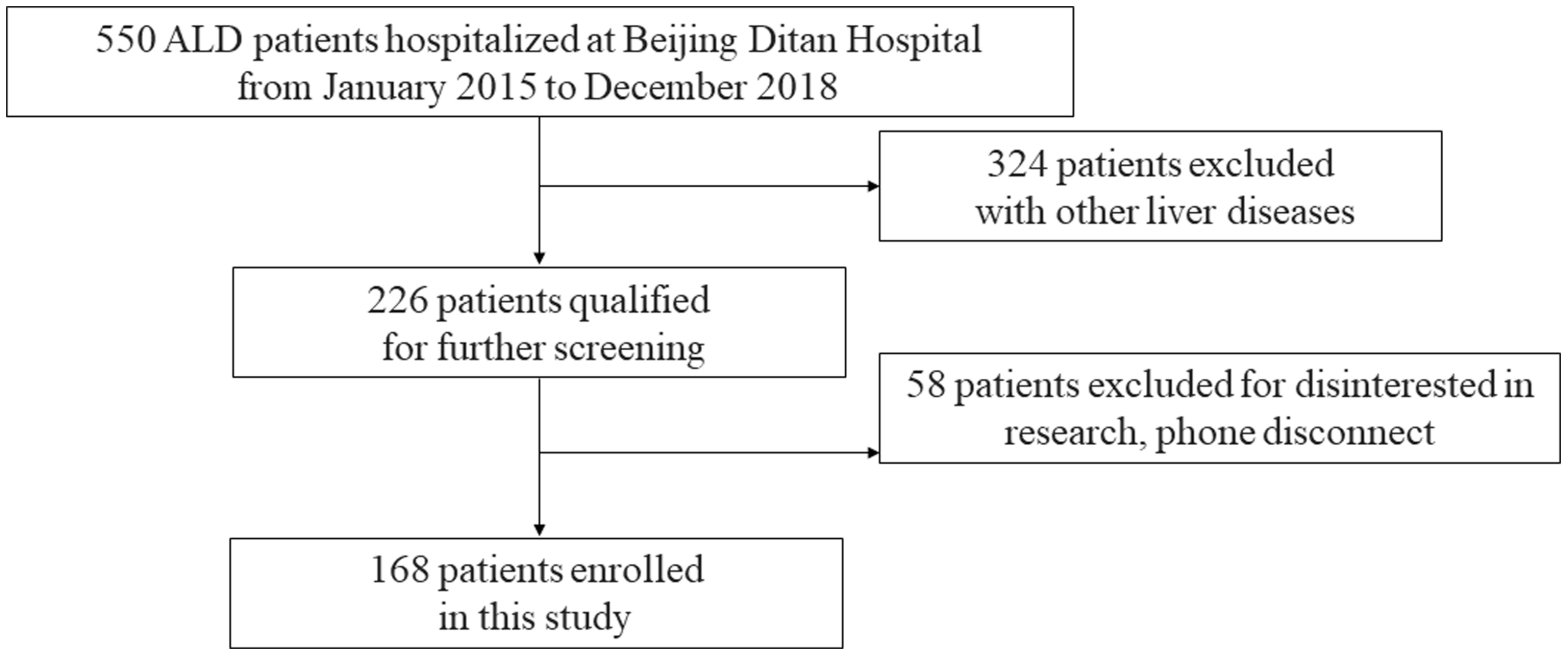
Flowchart of the study’s design.

Consecutive adult patients with ARLD hospitalized in the Department of Hepatology of Beijing Ditan Hospital between January 2015 and December 2018 were retrospectively enrolled. ARLD was diagnosed in accordance with the EASL Clinical Practice Guidelines for the Management of Alcohol-Related Liver Disease (18). In particular, a diagnosis of ARLD is suspected upon documentation of regular alcohol consumption of >20 g/d in females and > 30 g/d in males, according to the EASL guidelines of ARLD (18). The exclusion criteria were (1) viral hepatitis, autoimmune liver disease, drug-induced liver injury, primary liver cancer, or other liver diseases; (2) previous liver transplantation; and (3) missing follow-up data. Among 550 hospitalized patients initially diagnosed with ARLD and screened using a hospital information system, 324 patients were excluded because of comorbidities with other liver diseases, 226 patients qualified for further screening, and 58 patients excluded for disinterested in research, phone disconnect. Eventually,168 patients were enrolled in this study and finished 24 months follow-up in this study (Figure 1). The data on baseline demographics included sex, age, smoking history, and recidivism; the laboratory tests included complete blood counts, coagulation tests, and liver and renal function tests. The tests for liver cirrhosis decompensation complications included ascites, hepatic encephalopathy, and esophageal–gastric variceal bleeding.

### 2.2. Ct scan and assessment of the L3-SMI

CT scans were performed using a multi-slice spiral CT scanner (Brilliance 256-slice spiral CT scanner, Philips Medical Systems, the Netherlands, or uCT780 256-slice spiral CT scanner, United Imaging Medical Systems, Shanghai, China) with a collimating reconstruction thickness of 1 mm and an interval of 1 mm, according to the standard operating procedures. All participants were placed in the supine position and instructed to hold their breath to reduce breathing and movement artifacts during scanning.

Two independent radiologists analyzed the CT images using SliceOmatic V5.0 software (Rev-8, Tomovision, Montreal, Quebec, Canada). In accordance with a previous study, muscle tissues were identified on CT images based on Hounsfield unit (HU) thresholds ranging from 29 to 150, and subcutaneous and intermuscular adipose tissues were identified using HU thresholds ranging from 30 to 190. In addition, tissues with HU thresholds ranging from 50 to 150 were considered visceral adipose tissues. The skeletal muscles at the L3 level include the psoas major, erector spinalis, quadratus psoas, external abdominal oblique, internal abdominal oblique on the right and left sides, and transverse abdominis. The software automatically calculated the sum of the relevant tissue area, the average density at the L3 level cross-section, the skeletal muscle area, visceral adipose tissue area, subcutaneous adipose tissue area, skeletal muscle density, visceral adipose tissue density, subcutaneous adipose tissue density, and intermuscular adipose tissue density. The L3-SMI, which represents muscle mass, was calculated by dividing the skeletal muscle area at the L3 level by the square of the patient’s height (cm^2^/m^2^). The L3-SMI has been suggested for assessing sarcopenia because of the ease of obtaining CT and magnetic resonance imaging data at the level of L3 and the muscles that are imaged, including the psoas muscle, erector spinae, lumbar muscle, transversal abdominis, external oblique muscle, and intra-abdominal oblique muscle (12, 19). Based on a previous study conducted in China (12), the cut-off value for the L3-SMI for the diagnosis of sarcopenia was 40.2 cm^2^/m^2^ in male patients and 31.6 cm^2^/m^2^ in female patients.

### 2.3. Patient follow-up

Patients were followed up every 3–6 months for 24 months using medical records or telephone interviews. The main study endpoint was death or liver transplantation at 24 months of follow-up. Alcohol abstinence and relapse were also assessed at each follow-up visit.

### 2.4. Statistical analysis

Continuous variables are described as the mean (±standard deviation) or median (interquartile range) and were assessed using the independent sample *t*-test or Mann–Whitney U test. Categorical variables are expressed as counts and percentages and were assessed using the chi-squared or Fisher’s exact test. The Kaplan–Meier method was used to develop a survival curve, which was compared using the log-rank test. Variables found significant in a univariate analysis were incorporated into a multivariate Cox proportional hazards regression analysis, which was used to identify independent prognostic factors. The cut-off values of the L3-SMI were determined using receiver operating characteristic (ROC) curve analysis with the Youden index. Statistical significance was set at *p* < 0.05. Statistical analysis was performed using SPSS 24.0 and R software (R Foundation for Statistical Computing, Vienna, Austria).

## 3. Results

### 3.1. Baseline characteristics of the patients

The baseline characteristics of the patients are summarized in Table 1. The cohort included 168 male participants aged 20–85 years. All patients were followed-up for 24 months or until a study endpoint was met; 38 (22.62%) patients died within 24 months. The average alcohol consumption of all patients was 134.64 ± 69.82 g/d.

**Table 1:** Baseline characteristics of the enrolled patients.

Variable	Survival (*n* = 130)	Death (*n* = 38)	*p* value
Clinical characteristic			
Age, year	55.25 ± 10.66	54.26 ± 11.59	0.625
Abstinence, *n*%	63 (48.5)	29 (76.3)	0.002*
Cirrhosis, *n*%	99 (76.2)	35 (92.1)	0.038*
Ascites, *n*%	71(54.6)	29 (76.3)	0.017*
Varices bleeding, *n*%	14 (10.8)	3 (7.9)	0.765
Encephalopathy, *n*%	15 (11.5)	9 (5.4)	0.060
Laboratory parameters			
WBC (10^9^/L)	4.90 (3.87–7.31)	4.85 (3.75–7.82)	0.949
Hb (g/L)	111.44 ± 29.20	110.52 **±** 23.16	0.840
PLT (g/L)	112.20 (69.45–152.00)	71.00 (54.98–130.25)	0.022*
ALT (U/T)	33.15 (22.03–56.00)	24.65 (16.48–36.33)	0.006*
AST (U/T)	60.20 (36.05–99.20)	55.35 (33.50–87.65)	0.619
TBil (μmol/L)	33.80 (16.20–68.25)	60.55 (22.75–128.20)	0.016*
ALB (g/L)	32.15 (27.60–37.43)	31.50 (26.80–34.93)	0.238
Cr (μmol/L)	62.50 (55.60–72.65)	69.50 (59.68–93.10)	0.006*
PT-INR	1.26 (1.09–1.46)	1.46 (1.23–1.80)	0.001*
Na (mmol/L)	139.55 (136.28–142.40)	138.18 **±** 4.19	0.006*
L3-SMI	43.73 ± 9.23	38.76 ± 7.84	0.003*
Sarcopenia	48 (36.9)	24 (63.16)	0.004*
Alcohol consumption (g/d)	133.00 ± 69.71	140.26 ± 70.84	0.835
Scoring system			
MELD	8.24 (5.05–13.95)	14.57 (8.29–19.65)	<0.001*
MELD ≥21, *n*%	7 (5.4)	6 (3.6)	0.035*
Maddery	15.02 (6.10–30.11)	29.24 (11.05–45.05)	0.001*
Maddery ≥32, *n*%	28 (21.5)	18 (47.4)	0.002*
Child-Pugh A, *n*%	33 (25.4)	4 (10.5)	0.073
Child-Pugh B/C, *n*%	97 (74.6)	34 (89.5)	0.073

Data are reported as counts and percentages, median ± standard deviation, or medians with 25th and 75th percentiles, respectively. WBC, white blood cell; Hb, hemoglobin; PLT, platelet count; ALT, alanine transaminase; AST, aspartate transaminase; TBil, total bilirubin; ALB, albumin; Cr, creatine; PT-INR, prothrombin time-international standardization ratio; L3-SMI, skeletal muscle index at the level of the third lumbar vertebra; MELD score, model for end-stage liver diseases score.

The baseline characteristics of the patients who died or had survived at 24 months are presented in Table 1. Thirty-six patients died of liver-related disorders, such as hepatic encephalopathy, upper gastrointestinal hemorrhage, and septic shock (36/38), one patient died of cerebral hemorrhage (1/38), and one of pulmonary embolism (1/38). Significant differences between the survival and death groups were identified in the relapse rate (48.5% vs. 76.3%, *p* = 0.002), baseline cirrhosis (76.2% vs. 92.1%, *p* = 0.038), ascites (54.6% vs. 76.3%, *p* = 0.017), platelet count (112.20 vs. 71.00, *p* = 0.022), alanine transaminase (33.15 vs. 24.65, *p* = 0.006), total bilirubin (TBil) (33.80 vs. 60.55, *p* = 0.016), creatinine (62.50 vs. 69.50, *p* = 0.006), prothrombin time -INR (PT-INR) (1.26 vs. 1.46, *p* = 0.001), serum Na (139.55 vs. 138.18, *p* = 0.006), MELD (8.24 vs. 14.57, *p* < 0.001), and Maddrey’s differential function (15.02 vs. 29.24, *p* = 0.001). There were no significant differences between the survival and death groups regarding age (55.25 ± 10.66 vs. 54.26 ± 11.59, *p* = 0.625), varices bleeding (10.8% vs. 7.9%, *p* = 0.765), and encephalopathy (11.5% vs. 5.4%, *p* = 0.06). In particular, more patients (20) comorbid with sarcopenia died at 24 months of follow-up compared with patients who survived (66.67% vs. 85.42%, *p* = 0.004).

### 3.2. Cumulative survival in patients with and without sarcopenia

**Figure 2:**
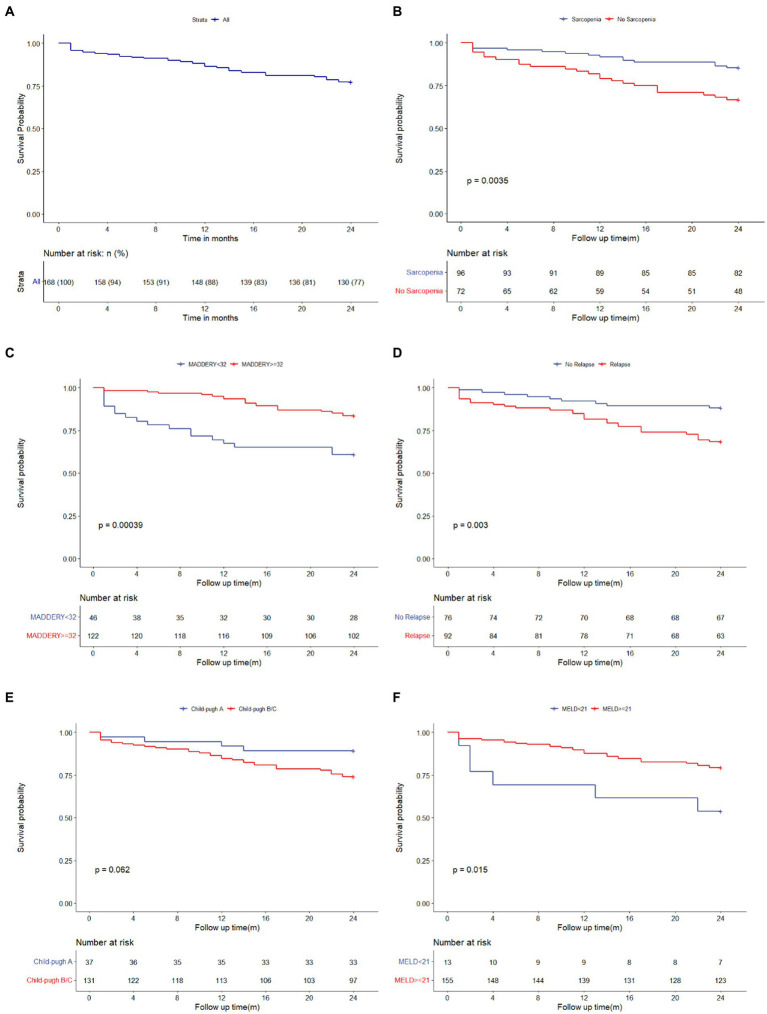
Kaplan–Meier estimate of the ARLD patients stratified. Kaplan–Meier curves for total survival of patients with ARLD **(A)**; Kaplan–Meier curves for survival of patients with sarcopenia and without sarcopenia **(B)**; Kaplan–Meier curves for survival of patients who followed abstinence and relapsed from abstinence **(C)**; Kaplan–Meier curves for survival of the patients with MELD score ≥ 21 and with MELD <21 **(D)**; Kaplan–Meier curves for survival of the patients with Maddery score ≥ 32 and with Maddery <32 **(E)**; Kaplan–Meier curves for survival of the patients with Child-Pugh A and with Child-Pugh B/C **(F)**. ARLD, alcohol-related liver disease.

A total of 72 patients had comorbid sarcopenia. The cumulative survival rates of patients at 3, 6, 12, and 24 months in this study were 94.05, 91.7, 86.31, and 77.38%, respectively (Figure 2A). Figure 2 illustrates the analysis of patient survival based on the various subgroups. The 24-month survival of patients with comorbid ARLD and sarcopenia was worse than that of patients without sarcopenia (*p* = 0.0035, Figure 2B). The 3-, 6-, 12-, and 24-month cumulative probabilities of survival in patients with ARLD comorbid sarcopenia were 90.28, 86.11, 79.17, and 66.67%, respectively. In comparison, they were 96.88, 95.83, 91.67, and 85.42%, respectively, in patients without sarcopenia.

The survival rate of patients with sarcopenia was worse than that of patients without sarcopenia at 24 months (66.67% vs. 85.42%, *p* = 0.004). In particular, the survival of patients who relapsed from abstinence was worse than that of patients who maintained abstinence (Figure 2c; *p* = 0.035). Furthermore, a comparison of the prognoses of patients with baseline MELD scores of <21 and ≥ 21 and Maddrey scores of <32 and ≥ 32 showed that the survival of patients with a baseline MELD score of ≥21 and Maddrey score of ≥32 was worse (Figures 2D,E; *p* = 0.015, *p* < 0.001, respectively). In addition, the 24-month survival of patients with ARLD and cirrhosis (ALC) was worse than that of patients without ALC.

During the clinical course in the follow-up period, there were 12 cases of hepatic encephalopathy, 8 cases of gastrointestinal hemorrhage, and 47 cases of ascites complications among the 72 patients who had comorbid sarcopenia. In comparison, there were 12 cases of hepatic encephalopathy (*p* = 0.507), 9 cases of gastrointestinal hemorrhage (*p* = 0.798), and 53 cases of ascites complications (*p* = 0.207) in the 96 patients without sarcopenia, and there was no significant difference between the two groups. Additionally, the rehospitalization of patients with sarcopenia was more than that of patients without sarcopenia at 24 months (40.70% vs. 51.50%, *p* = 0.327).

### 3.3. Cox regression analysis of independent prognostic factors

A univariate Cox analysis was first performed using the enrolled patient data, identifying that the factors most related to ARLD were abstinence, cirrhosis, ascites, platelet count, TBil, creatinine, the PT-international standardization ratio, Na^+^, the L3-SMI, sarcopenia, MELD score ≥ 21, and Maddrey score ≥ 32 (Table 2). These variables were entered into a multivariate analysis model, except for the L3-SMI, MELD, and Maddrey scores, since the L3-SMI was included in the diagnosis of sarcopenia, and the MELD and Maddrey scores included other variables. The analysis revealed that sarcopenia, abstinence, and baseline creatinine levels were independent factors for the 24-month prognoses of patients with ARLD, with hazard ratios and 95% confidence intervals (CI) of 2.022 (1.025–3.991), 0.275 (0.122–0.617), and 1.018 (1.008–1.027), respectively (Table 3).

**Table 2:** Univariable analysis of ARLD patients.

Variable	HR	95% CI	*p* value
Age	0.993	0.964–1.022	0.631
Abstinence	0.341	0.162–0.721	0.005*
Cirrhosis	3.216	0.989–10.459	0.052
Ascites	2.372	1.123–5.013	0.024*
Varices bleeding	0.727	0.224–2.363	0.596
Encephalopathy	2.131	1.008–4.504	0.048*
WBC (10^9^/L)	1.028	0.936–1.129	0.567
Hb (g/L)	0.998	0.987–1.010	0.776
PLT (g/L)	0.997	0.992–1.001	0.139
ALT (U/T)	0.995	0.987–1.004	0.268
AST (U/T)	0.999	0.996–1.001	0.428
TBil (μmol/L)	1.003	1.001–1.006	0.022*
ALB (g/L)	0.957	0.908–1.009	0.104
Cr (μmol/L)	1.018	1.010–1.027	<0.001*
PT-INR	2.201	1.294–3.744	0.004*
L3-SMI	0.943	0.907–0.980	0.003*
Sarcopenia	2.560	1.324–4.952	0.005*
MELD ≥21	2.805	1.171–6.714	0.021*
Maddery ≥32	2.981	1.575–5.643	0.001*
Child-Pugh B/C	0.389	0.138–1.097	0.074

*p value <0.05 was considered significant. CI, confidence interval; AH, alcoholic hepatitis; WBC, white blood cell; Hb, hemoglobin; PLT, platelet count; ALT, alanine transaminase; AST, aspartate transaminase; TBil, total bilirubin; ALB, albumin; Cr, creatine; PT-INR, prothrombin time-international standardization ratio; MELD score, model for end-stage liver diseases score.

**Table 3:** Cox regression analysis for identifying independent prognostic factors.

Variable	HR	95% CI	*p* value
Abstinence	0.275	0.122–0.617	0.002*
Ascites	1.942	0.861–4.382	0.110
Encephalopathy	1.316	0.568–3.048	0.522
TBil (μmol/L)	1.001	0.998–1.004	0.557
Cr (μmol/L)	1.018	1.008–1.027	<0.001*
PT-INR	1.332	0.617–2.872	0.465
Sarcopenia	2.022	1.025–3.991	0.042*

*p value <0.05 was considered significant. TBil, total bilirubin; Cr, creatine; CI, confidence interval; PT-INR, prothrombin time-international standardization ratio.

### 3.4. Analysis of the L3-SMI for predicting the prognoses of patients with ARLD

**Figure 3:**
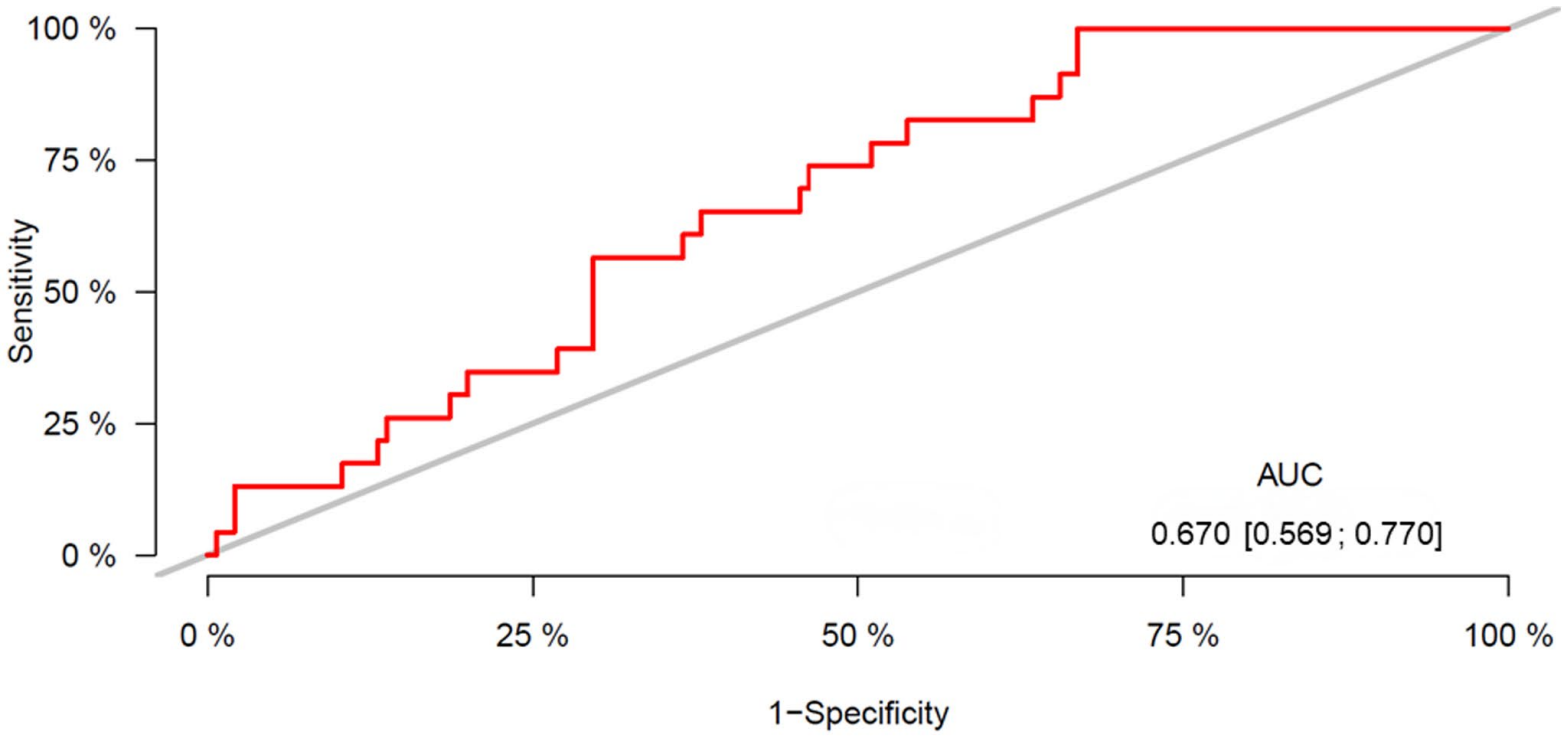
ROC curve for the L3-SMI. Area under the curve (AUC) = 0.67; confidence interval 0.569–0.770; cut-off 40.00; sensitivity 64.6% and specificity 63.2%. ROC, receiver operating characteristic; SMI, skeletal muscle index.

ROC curve analysis was performed to evaluate the performance of the L3-SMI in predicting the prognoses of patients with ARLD. The area under the ROC curve value of the L3-SMI for defining sarcopenia in the entire study group of males was 0.67 (95% CI: 0.569–0.770) (Figure 3), and the cut-off value of the L3-SMI in males was 40.0 cm^2^/m^2^ with 64.6% sensitivity and 63.2% specificity.

## 4. Discussion

Numerous studies have claimed that sarcopenia is highly and independently associated with liver diseases (7, 21). Liver diseases, especially cirrhosis and end-stage liver disease, are characterized by decreased appetite, insufficient energy intake, impaired nutrient digestion and/or malabsorption, and decreased physical activity (10). In addition, patients with cirrhosis and end-stage liver disease commonly have abnormal glycogen metabolism; therefore, more skeletal muscle proteins must be decomposed to provide amino acids for gluconeogenesis, resulting in increased skeletal muscle loss (22, 23). Hormone deficiencies (lower levels of insulin-like growth factor 1, vitamin D, and testosterone), inflammatory cytokine activation [upregulated expression of tumor necrosis factor-α (TNF- α) and interleukin-6 (IL-6)], and other metabolic disorders in cirrhosis also may lead to decreased skeletal muscle protein synthesis and increased autophagy (24).

There have been seldom reports examining patients with ARLD comorbid with sarcopenia and their prognosis in China. Among Chinese patients with cirrhosis, 29.1% of male patients were comorbid with sarcopenia; however, only 61 patients with alcohol-related cirrhosis were analyzed (6). Mindie reported that 35% of patients with cirrhosis were comorbid with sarcopenia, but the incidence of patients with ARLD comorbid was greater than that of cirrhosis from other causes: up to 50% (7). Our cohort enrolled 168 male patients with ARLD and found that 42.86% (72/168) were comorbid with sarcopenia, consistent with previous reports. Alcohol and its metabolites can induce sarcopenia in patients with ARLD *via* a variety of biological mechanisms (20, 25). Ethanol inhibits the mammalian target-of-rapamycin protein complex 1 (mTOR) and mTOR signaling targets, reducing protein synthesis and increasing autophagy. In patients with ARLD and cirrhosis, protein synthesis was deficient, resulting in “anabolic resistance (26, 27).” In addition, ethanol intake reduces hepatocyte ureagenesis and increases muscle ammonia transporter RhBG expression and muscle ammonia levels.

The direct effects of ethanol are synergistic with increased ammonia absorption in generating dysregulated skeletal muscle proteostasis and signaling perturbations, resulting in sarcopenia (28). Ethanol also induces the expression of inflammatory cytokines, including TNFα and IL-6, which accelerate skeletal muscle protein degradation through the ubiquitin-protease pathway (29). Ethanol promotes mitochondrial dysfunction in skeletal muscle, reduces protein synthesis, and increases autophagy by targeting mitochondrial reactive oxygen species (30). These factors may have caused the observed higher rate of sarcopenia in patients with ARLD.

In our cohort, comorbid sarcopenia was associated with a two-fold risk of 24-month mortality in male patients with ARLD in mainland China. This finding is consistent with a previous study that showed a two-fold greater risk of global mortality in patients with cirrhosis (31–33). This study also identified abstinence as an independent prognostic factor for patients with ARLD. Identifying patients at a high risk of recidivism during the follow-up period is crucial for early relapse intervention in patients with ARLD, and multidisciplinary intervention to maintain abstinence is needed as a crucial part of ARLD management (34, 35). In addition, we also found that kidney dysfunction was an independent prognostic factor in patients with ARLD. ARLD, especially alcoholic hepatitis and alcohol-related cirrhosis, is closely related to hemodynamic disorders, including portal hypertension and systemic inflammatory response syndrome, which place patients at high risk for kidney dysfunction. The prognostic significance of kidney dysfunction is well recognized; serum creatinine or urea is included in several ARLD prognostic scores, including the MELD score, ABIC, GAHS, and the Lille model.

The present study used the L3-SMI to diagnose sarcopenia. Skeletal muscle mass, as measured by CT, represents whole-body muscle mass and can be linked positively with the patient’s grip strength, walking speed, and other physical performance metrics; thus, it represents the patient’s overall sarcopenia while also representing the body’s muscle mass (36). However, the L3-SMI cut-off value for diagnosing sarcopenia was not determined in Chinese patients until the multicenter report by Kong et al.; the cut-off value for males was 40.2 cm^2^/m^2^. In our study, we used this cut-off value to define sarcopenia. In addition, we verified the L3-SMI cut-off value for prognostic prediction in patients with ARLD. The cut-off value for L3-SMI for males in mainland China was 40.0 cm^2^/m^2^, consistent with Kong et al.’s report. However, the threshold values of different detection methods used in studies on patients with ARLD-comorbid sarcopenia urgently need to be centralized. The relevance of L3-SMI in the diagnoses of sarcopenia in patients with ARLD had previously not been investigated.

The current study found that the overall 24-month survival rate of Chinese patients with ARLD was 77.38%, with an overall sarcopenia prevalence of 42.86%, consistent with other studies (7, 37). This study is the first to use sarcopenia diagnosed using the L3-SMI to predict the prognoses of Chinese patients with ARLD. The prognosis of patients with ARLD and comorbid sarcopenia is an extremely serious and widespread phenomenon that requires adequate attention in the clinical setting. Consequently, in addition to managing abstinence and complications, attention should focus on managing and evaluating sarcopenia in patients with ARLD, particularly those with severe alcoholic hepatitis. Our study showed that sarcopenia is an independent prognostic factor in patients with ARLD.

This study has several limitations. First, because this study was performed in a single center and retrospectively, an external validation cohort and additional measure of muscle strength/function were not available. Second, the participants were all male; thus, these data cannot be generalized to females, and further research involving female patients is needed. As a retrospective study, 58/226 patients were excluded for phone disconnect or personal reasons during screening period, which might cause a selection bias. However, all patients enrolled finished 2 years follow-up. Nevertheless, in China, this study is the first to be conducted on patients with ARLD-comorbid sarcopenia, and the analysis revealed significant results. A further multicenter prospective study with a larger sample size is currently in progress to validate our study results.

## Data availability statement

The raw data supporting the conclusions of this article will be made available by the authors, without undue reservation.

## Ethics statement

Ethical approval was not required for the studies involving humans because the study used retrospective anonymised data. The studies were conducted in accordance with the local legislation and institutional requirements.

## Author contributions

SY accepts full responsibility for the conduct of the study, has access to the data, and has control of the decision to publish. YZ proposed the concept, contributed to the study design, wrote the manuscript, and performed statistical analysis. CC and LW contributed to the study design and performed statistical analysis. FD and MQ contributed to data collection. All authors contributed to the article and approved the submitted version.

## Funding

This study was supported by the High-level public health talent cultivation project (XKGG-02-30), Beijing Municipal Administration of Hospitals Incubating Program (PX2022071) and the Tianqing Foundation of Chinese Foundation for hepatitis prevention and control (TQGB20210050).

## Conflict of interest

The authors declare that the research was conducted in the absence of any commercial or financial relationships that could be construed as a potential conflict of interest.

## Publisher’s note

All claims expressed in this article are solely those of the authors and do not necessarily represent those of their affiliated organizations, or those of the publisher, the editors and the reviewers. Any product that may be evaluated in this article, or claim that may be made by its manufacturer, is not guaranteed or endorsed by the publisher.
